# Effects of Integrating Wearable Activity Trackers With a Home-Based Multicomponent Exercise Intervention on Fall-Related Parameters and Physical Function in Older Adults: Randomized Controlled Trial

**DOI:** 10.2196/64458

**Published:** 2025-05-08

**Authors:** Yejin Kim, Kyung Hee Park, Hye-Mi Noh

**Affiliations:** 1 Department of Medical Sciences, College of Medicine, Hallym University, Chuncheon, Republic of Korea; 2 Department of Family Medicine, Hallym University Sacred Heart Hospital, Hallym University, 22, Gwanpyeong-ro 170beon-gil, Anyang, 14068, Republic of Korea, 82 313803805

**Keywords:** fear of falling, physical function, home exercise, multicomponent exercise, older adults, physical activity intervention, step counts, wearable activity tracker, exercise, intervention, gerontology, geriatrics, older person, aging, activity tracker, physical activity, self-monitoring, randomized controlled trial, wearable technology, fall, falling

## Abstract

**Background:**

Older adults with a history of falling often encounter challenges in participating in group exercise programs. Recent technological advances, such as activity trackers, can potentially enhance home-based exercise programs by providing continuous physical activity monitoring and feedback.

**Objective:**

The aim of the study is to explore whether integrating wearable activity trackers with a home-based exercise intervention is effective in reducing fear of falling and improving physical function in older adults.

**Methods:**

This was a 12-week, parallel-group, randomized controlled trial involving 30 older adults (≥60 years) with a history of falling. Participants were randomly assigned in a 1:1 ratio to either a group combining an activity tracker with a home-based multicomponent exercise intervention, which included in-person exercise sessions, exercise videos, and objective feedback via phone calls (AT+EX group) or to a group using the activity tracker only for self-monitoring (AT-only group). The primary and secondary outcomes included fall-related parameters (fear of falling assessed by the Activities-Specific Balance Confidence [ABC] and the Falls Efficacy Scale-International [FES-I] scales), depression (Short Geriatric Depression Scale), cognition (Montreal Cognitive Assessment), physical function (grip strength, Short Physical Performance Battery, Timed Up and Go [TUG] test, and 2-Minute Step Test), and body composition. Changes in the average daily step count were monitored and analyzed.

**Results:**

Overall, 28 (mean age 74.0, SD 6.4 years; n=23, 77% female) participants completed the 12-week follow-up period (28/30, 93%). In the activity tracker and exercise group (AT+EX group), significant improvements were observed in fear of falling (15.5 points of ABC: *P*=.002; –5.1 points of FES-I: *P*=.01). The activity tracker alone group (AT-only group) also showed a significant improvement in FES-I score (–5.5 points: *P*=.01). Physical function significantly improved in the AT+EX group (1.1 points of Short Physical Performance Battery: *P*=.004; –1.4 seconds of TUG; *P*=.008; and 26.7 steps of 2-Minute Step Test: *P*=.001), whereas the AT-only group showed significant improvement only in the TUG test (–1.3 seconds: *P*=.002). However, no significant between-group differences were observed in the ABC score, FES-I score, or physical function. Despite no significant increase in daily step counts, both groups maintained close to 10,000 steps per day throughout the 12 weeks.

**Conclusions:**

Both groups showed improvements in the FES-I and TUG test scores without significant between-group differences. Wearable technology, with or without an exercise intervention, seems to be an effective tool in reducing the fear of falling and improving physical function in older adults susceptible to falls.

## Introduction

Falls are a significant health issue in older adults and are among the most prevalent geriatric syndromes [1]. The consequences of falls can be severe, resulting in injuries such as fractures, bleeding, and disability, leading to increased health care costs [2]. Falls increase the likelihood of nursing home admissions among older adults and are a leading cause of accidental death [3]. According to the Centers for Disease Control and Prevention, 27.6% of individuals aged >65 years in the United States reported falling during the previous year, and 38,742 (78.0 per 100,000) died from unintentional falls in 2020 [4]. In Korea, the 2017 National Survey of Korean Elderly reported that 15.9% of older adults experienced falls in the past year, with an average of 2.1 falls per person [5]. A history of falls is a significant risk factor for future falls, as approximately 1 in 2 individuals who have experienced falls may experience recurrent falls [6]. Additionally, the fear of falling, characterized by anxiety concerning falling or a loss of confidence in certain activities, leads to decreased physical activity and an increased risk of further falls [7].

Numerous studies have demonstrated the effectiveness of exercise interventions in preventing falls and improving physical function in older adults [8,9]. Each type of exercise (ie, resistance, aerobic, and balance training) has specific benefits, and incorporating high-challenge balance training in exercise programs is particularly emphasized for fall prevention [9,10]. Resistance training is reportedly effective in preventing age-related decline in muscle function, especially with high-intensity training [11]. Clinical guidelines from leading health and geriatric organizations recommend that older adults participate in multicomponent exercise programs, including balance and resistance training, as well as aerobic and flexibility activities, to improve physical function and reduce the risk of falls [12-14].

Effective doses and consistent exercise are crucial for maintaining health in older adults [9]. However, adherence to home-based exercises is typically lower compared to that for community-based exercises, and self-reported physical activity levels often exceed actual levels [15]. Although many group exercise programs have been conducted for older adults in the community, the onset of the COVID-19 pandemic has made it challenging to continue group exercise sessions [16]. Moreover, factors, such as low confidence in physical activity and self-efficacy, further hinder participation in group exercises [17]. Older adults also face restrictions in participating in physical activity programs because of physical and mental health issues (ie, risk of injury, joint pain, and fear of falling) and structural and organizational barriers (ie, access to facilities, limited infrastructure, and cost) [18].

Recently, various wearable devices have been developed and are increasingly used as popular devices for monitoring physical activity. Activity trackers provide individuals with objective measures of physical activity levels and enable health professionals to offer precise feedback and sustained support [19]. The use of activity trackers, either as the primary element or within a broader behavioral intervention, effectively increases physical activity participation [20]. Moreover, integrating activity trackers with traditional intervention components is more effective than using an activity tracker alone [21].

Despite these advancements, there is a lack of research on the integration of activity trackers with other multifaceted interventions for falls in older adults [22,23]. Furthermore, to date, no study has evaluated the effect of combining activity trackers with home-based multicomponent exercises in older adults. Therefore, our aim was to assess the effectiveness of integrating activity trackers with home-based interventions in older adults with a history of falls. We hypothesized that combining an activity tracker with a home-based multicomponent exercise intervention (activity tracker and exercise group [AT+EX group]) would result in greater improvements in fall-related parameters and physical function compared to using an activity tracker alone (activity tracker alone group [AT-only group]).

## Methods

### Study Design

This 12-week, parallel-group, randomized controlled trial involved older adults aged ≥60 years with a history of falls and COVID-19 in Anyang, Gyeonggi-do, South Korea. Participants were randomly assigned in a 1:1 ratio to either the AT+EX or AT-only group. The AT+EX group received multicomponent exercise sessions, exercise videos, and objective feedback from an activity tracker, whereas the AT-only group received activity trackers without additional interventions. A CONSORT (Consolidated Standards of Reporting Trials) checklist was completed to guide the design, analysis, and reporting of trial findings (Checklist 1).

### Participants

Participants were recruited through poster advertisements, flyers, word-of-mouth referrals, and visits to local senior welfare centers. Eligible individuals met the following criteria: (1) aged ≥60 years, (2) history of COVID-19 infection within 1 year from their first study visit, (3) experience with falls, (4) ownership of a smartphone compatible with wearable devices, (5) ability to walk independently without assistance, and (6) voluntary decision to participate in the study with a signed informed consent form before participation. The exclusion criteria were as follows: (1) diagnosis of cognitive decline or dementia and (2) contraindications to exercise, including myocardial infarction within the last 3 months, unstable angina pectoris, uncontrolled diabetes, or musculoskeletal disease that prevented exercise.

### Sample Size Calculation and Randomization

According to Giné-Garriga et al [24], the difference in Activities-Specific Balance Confidence (ABC) scale scores between the functional circuit training group and the routine daily activity control group was 6.65 points [24]. Based on this score difference, assuming a type 1 error of 0.05, statistical power of 80%, and an anticipated dropout rate of 20%, 26 (13 per group) participants were required. Therefore, 30 participants were recruited, 15 of whom were assigned to each group. The research team recruited the participants for the trial. A person who was not involved in the trial carried out randomization. After completing the baseline surveys, the participants were randomly assigned to the intervention (AT+EX group) or control group (AT-only group) using block randomization and randomly selected block sizes of 2, 4, or 6. Blinded assessors evaluated fall-related parameters, body composition, and the data of mean daily step counts from activity trackers.

### Intervention

All participants were provided with a wearable activity tracker (HL5, Seven Elec) and its corresponding application. The participants received an individual information session on how to use, pair, and charge the activity tracker during their first visit and were advised to wear the tracker on their wrists throughout the 12-week study period. The activity tracker data were paired and synchronized with the Health & You app on a mobile device. The daily step goal was set at 10,000 steps, and adjustments were made only if necessary, based on the participant’s physical ability and current activity levels. The step goals remained consistent throughout the 12 weeks. Participants could track their daily steps via the activity tracker and mobile phone app and were required to synchronize their device with the app at the end of each day. The researcher could view the participants’ activity tracker data on the admin page.

The AT-only group received education on the use of an activity tracker and educational materials for general fall prevention exercises. This group focused on self-monitoring using a wearable device to increase daily physical activity and did not receive any exercise interventions or feedback. Participants in the AT-only group visited the clinic at baseline and after 12 weeks.

The participants randomized to the AT+EX group received an activity tracker and participated in a home-based fall exercise intervention program. The exercise intervention included 4 in-person sessions (weeks 1, 3, 6, and 9) conducted at either a hospital or a senior welfare center. Each session was conducted by a certified exercise trainer and at least 1 assistant, with 1‐6 participants. The exercise protocol was specifically designed to prevent falls in older adults, including balance, resistance, aerobic, and multitask training, which are effective in preventing falls [9,10,13]. Each 1-hour exercise session consisted of 5 minutes of stretching, 15 minutes of balance training, 15 minutes of strength training, 15 minutes of gait and aerobic exercise, and 10 minutes of multitask training. Over the 12-week intervention period, the exercise protocol consisted of 3 phases (first, second, and third stages), with a gradual increase in intensity between each phase. The detailed exercise program for each level is described in Multimedia Appendix 1. All participants in the AT+EX group were provided exercise equipment (step boxes, TheraBands, and yoga mats). To support consistent exercise at home, the participants received exercise videos that followed the same protocol as the in-person sessions. They were asked to exercise at least thrice a week to meet the physical activity recommendations for older adults [13]. Throughout the 12-week study period, the participants received 6 physical activity counseling phone calls (weeks 2, 4, 5, 7, 10, and 11). Phone calls were conducted by a researcher (YK) with a master’s degree in exercise physiology, who followed the same protocol for all participants. Each call included feedback on physical activity levels derived from their activity trackers, aiming to motivate participants to initiate and sustain their physical activity and exercise routines. Additionally, the participants were asked concerning their recent health status, barriers to participation in the study, and any issues with using their wearable devices.

### Fall-Related Parameters

The primary outcomes were fall-related parameters, including fear of falling, depression, and cognitive function, all evaluated using the Korean version and measured at baseline and 12 weeks. Fear of falling was measured using the ABC scale and Falls Efficacy Scale-International (FES-I). The ABC scale was used to assess the individuals’ confidence in performing various activities without losing balance or falling [25]. The questionnaire included 16 items, with scores ranging from 0%=no confidence at all to 100%=completely confident. The overall score was calculated by averaging the scores for all 16 items. The FES-I measures self-efficacy and fear of using 16 items, each rated on a scale of 1=not at all concerned to 4=very concerned. The total score ranged 16‐64 points, with higher scores indicating a greater fear of falling [26]. Depression, which is closely related to the fear of falling among older adults, was assessed using the Short Geriatric Depression Scale (SGDS), a reliable and valid self-administered questionnaire designed to screen for depression in older adults [27]. The SGDS comprises 15 yes-or-no questions, with higher scores indicating a more depressed mood. The Montreal Cognitive Assessment, a tool for screening mild cognitive impairment, was used to assess cognitive function. Scores range 0‐30 points, with scores of ≤22 points indicating mild cognitive impairment [28].

### Physical Function

Physical function was assessed at baseline and 12 weeks by the same assessor using the same protocol. Grip strength is a key measure used to assess overall health and muscle strength, particularly in older adults [29]. This was assessed using a calibrated digital dynamometer (Takei 5401-C; Takei Scientific Instruments; in kilograms). Individuals were asked to squeeze the dynamometer with maximum effort, performing 3 alternating measurements on each side. The highest values obtained from these trials were used in this study. According to the Asian Working Group for Sarcopenia, low grip strength is defined as <28 kg for male and <18 kg for female participants [30]. The Short Physical Performance Battery (SPPB) is used to assess lower-extremity function and physical performance in older adults [31]. The SPPB includes 3 components: balance tests (side-by-side, semitandem, and tandem stances), a gait speed test (4-m distance), and a 5-time sit-to-stand test. Each component was scored 0‐4 points, with a total score ranging 0‐12 points. Higher scores indicate better performance. A change of ≥1 point in the SPPB score was considered a clinically important difference [32]. The Timed Up and Go (TUG) test was used to assess balance and mobility. Each individual was asked to rise from a chair and walk 3 m, turn around, walk back to the chair, and sit down. The time taken to complete the task was measured in seconds. A shorter time indicated better mobility. Scores of ≥13.5 seconds indicate a high risk of falling [33]. The 2-Minute Step Test (2MST) was used to assess aerobic capacity and endurance as an alternative aerobic endurance test in environments with limited space [34]. The participants were instructed to march in place for 2 minutes, lifting their knees midway between the patella and the iliac crest as many times as possible. Performance was measured by counting the number of right-sided steps completed within the 2-minute period. Higher step counts indicated better aerobic endurance.

### Body Composition

Anthropometric measurements were obtained from the participants at baseline and at 12 weeks while wearing light clothes and no shoes, except for height, which was measured only at baseline. Height measurements were recorded to the nearest 0.1 cm using a stadiometer, while weight measurements were recorded to the nearest 0.1 kg with a digital scale. BMI was calculated by dividing weight by height squared (kg/m²). Body composition was assessed using a dual-energy x-ray absorptiometry scanner (Lunar Prodigy Advance; GE Healthcare Technologies). The total fat, total lean, and appendicular muscle masses were determined. Appendicular muscle mass was calculated as the sum of the lean mass in both arms and legs divided by the square of height (kg/m²).

### Demographics

Basic demographic factors were assessed at baseline and included age, sex, marital status (single, married, widowed, or divorced), education level (college or higher, high school, middle school, or elementary school), occupation (working or not working), number of falls in the previous year, and the presence of comorbidities (eg, hypertension, diabetes, cancer, and other chronic diseases, such as cardiovascular and musculoskeletal disorders).

### Mean Step Counts

The mean daily step count was assessed using data from activity trackers and was calculated based on the number of steps recorded over a specific week. The baseline step count was defined as the mean number of daily steps in the first week (days 1‐7) after the initial visit. Subsequent steps were recorded as the daily average steps between each exercise session (weeks 1, 3, 6, and 9), and the final average steps were recorded from the fourth exercise session to the final visit (week 12). For the AT-only group, average daily step counts were recorded during the same period as those for the AT+EX group, specifically at weeks 1, 3, 6, 9, and 12. Participants with <50% wear days during these periods were excluded from the analysis. Additionally, days with <500 steps were considered nonwear days and excluded from the step count calculations [35].

### Statistical Analysis

Continuous variables are presented as means and SDs, while categorical variables are presented as frequencies and percentages. The baseline characteristics of the AT+EX and AT-only groups were examined using independent 2-tailed *t* tests for continuous variables and chi-square tests for categorical variables. Outcome data, including all available data, were analyzed using the intention-to-treat approach. When data were missing due to dropouts of participants, we used the last observation carried forward method to impute missing values. Initially, independent 2-tailed *t* tests or Mann-Whitney *U* test were used to determine whether there were any significant baseline differences between the 2 groups in fall-related outcomes, physical function, and body composition. Subsequently, to compare changes from baseline to 12 weeks (Δ values) between the groups, independent 2-tailed *t* tests or Mann-Whitney *U* tests were conducted. Paired 2-tailed *t* tests or Wilcoxon signed rank tests were used to evaluate within-group changes from baseline to 12 weeks. The change in average daily step count over the 12-week study period was analyzed using a mixed-effects model for repeated measures to account for within-participant correlations. The change in the average daily step count was calculated by subtracting the mean step count at baseline from that at each subsequent visit (weeks 3, 6, 9, and 12). Subgroup analyses were conducted for individuals with SPPB scores ≤9 (low physical performance). All statistical analyses were conducted using SPSS statistical software (version 26.0; IBM Corp) and R software (version 4.2.0; R Foundation for Statistical Computing), with statistical significance set at *P*<.05.

### Ethical Considerations

This study was approved by the institutional review board of Hallym University Sacred Heart Hospital (HALLYM 2022-10-020) and conducted in accordance with the tenets of the Declaration of Helsinki. The study was registered with the Clinical Research Information Service on February 23, 2023 (KCT0008230). All participants provided written informed consent after receiving a comprehensive explanation of the study objectives, procedures, and their right to withdraw at any time. To ensure confidentiality, all data were anonymized and restricted to authorized researchers. Participants received a transportation allowance of US $34.97 as compensation.

## Results

### Baseline Characteristics

**Figure 1.**
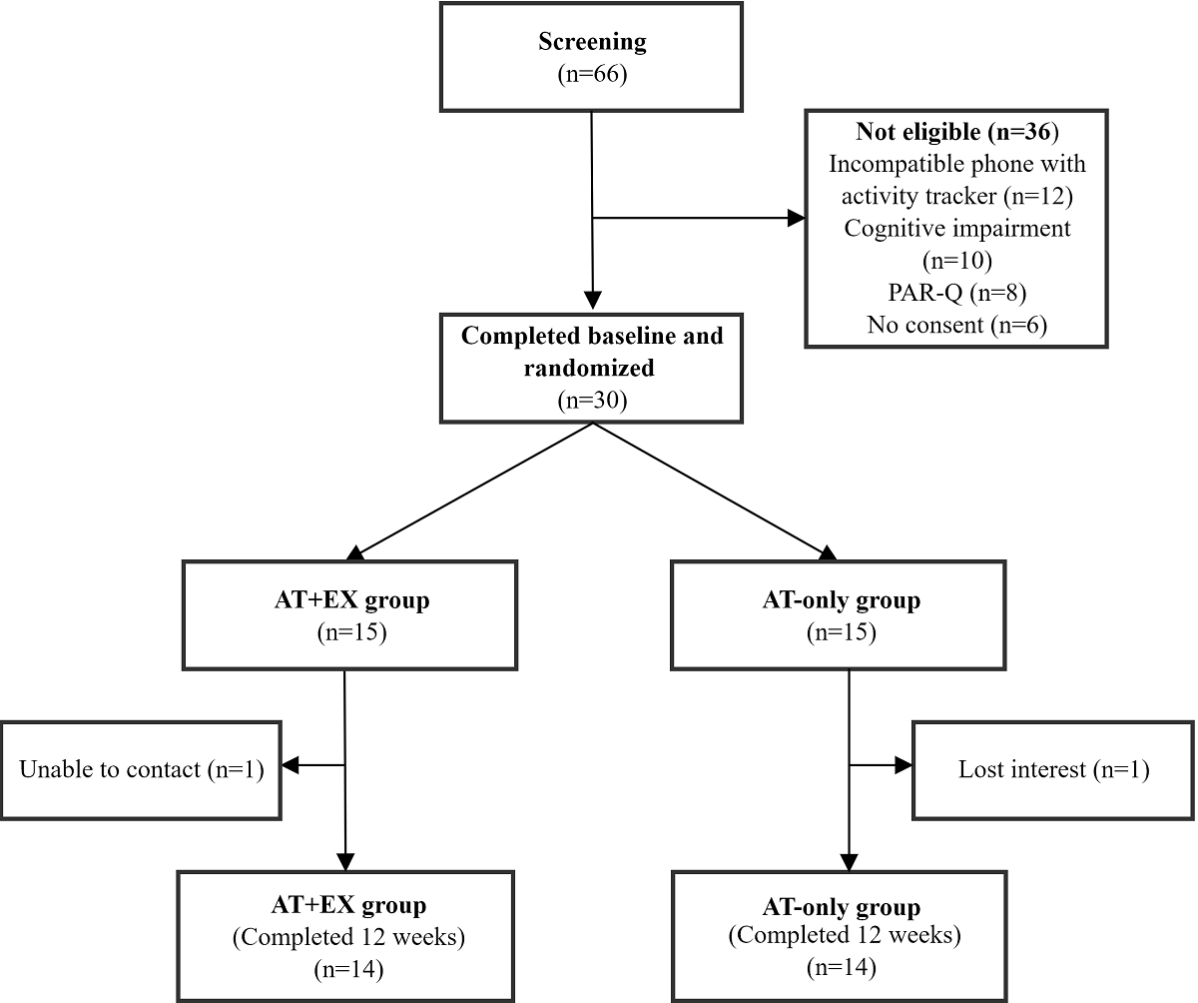
Participant flowchart. AT+EX group: activity tracker and exercise group; AT-only group: activity tracker alone group; PAR-Q: Physical Activity Readiness Questionnaire.

Recruitment occurred from February to August 2023 until the target sample size was achieved. Overall, 30 participants were enrolled and randomly assigned to either the AT+EX or the AT-only group (Figure 1). The baseline characteristics of the study participants are presented in Table 1. The mean age of the participants was 74.0 (SD 6.4) years, and 77% (23/30) were female participants. A significant difference was observed in body weight, with participants in the AT+EX group (mean 55.1, SD 8.6 kg) having a significantly lower body weight compared to those in the AT-only group (mean 62.0, SD 9.1 kg; *P*=.04). No significant differences were observed in BMI, marital status, educational level, occupation, number of falls during the previous year, or comorbidities between the groups.

**Table 1.** Baseline characteristics of the participants.

	Total (n=30)	AT+EX group^a^ (n=15)	AT-only group^b^ (n=15)	*P* value^c^
Age (years), mean (SD)	74.0 (6.4)	73.5 (5.3)	74.6 (7.4)	.64
Sex, n (%)				>.99
Male	7 (23)	3 (20)	4 (27)	
Female	23 (77)	12 (80)	11 (73)	
Anthropometrics, mean (SD)				
Height (cm)	156.4 (9.0)	155.1 (8.7)	157.7 (9.4)	.45
Weight (kg)	58.6 (9.4)	55.1 (8.6)	62.0 (9.1)	.04
BMI (kg/m^2^)	23.9 (3.0)	22.8 (2.7)	24.9 (3.1)	.06
Marital status, n (%)				.68
Single	1 (3)	0 (0)	1 (7)	
Married	24 (80)	12 (80)	12 (80)	
Widowed	3 (10)	2 (13)	1 (7)	
Divorced	2 (7)	1 (7)	1 (6.)	
Education level, n (%)				.15
Less than elementary school	8 (27)	3 (20)	5 (33)	
Middle school	7 (23)	2 (13)	5 (33)	
High school	8 (27)	4 (27)	4 (27)	
College and higher	7 (23)	6 (40)	1 (7)	
Occupation, n (%)				.65
Working	6 (20)	4 (27)	2 (13)	
Not working	24 (80)	11 (73)	13 (87)	
Number of falls during the previous year, mean (SD)	2.4 (3.5)	2.9 (4.8)	1.9 (1.5)	.25
Comorbidity, n (%)				.83
0	6 (20)	4 (27)	2 (13)	
1	9 (30)	4 (27)	5 (33)	
≥2	15 (50)	7 (47)	8 (53)	

a AT+EX group: activity tracker and exercise group.

b AT-only group: activity tracker alone group.

c *P* values represent the differences between the AT+EX group and the AT-only group. Independent 2-tailed *t* tests were used for continuous variables and chi-square tests for categorical variables.

### Fall-Related Outcomes

The intention-to-treat analyses of the primary outcomes, physical function, and body composition are presented in Table 2. Changes in fear of falling, assessed using the ABC and FES-I scores, showed significant improvement in the AT+EX group (*P*=.002 and *P*=.01, respectively). The AT-only group also showed improvements in the FES-I score (*P*=.01); the increase in the ABC score was only marginally significant (*P*=.07). Depression levels, assessed using the SGDS, significantly improved in both the AT+EX (*P*=.006) and AT-only groups (*P*=.02). Cognitive function, as measured by the Montreal Cognitive Assessment, showed no significant change in both the AT+EX (*P*=.10) and AT-only groups (*P*=.49). No significant differences were detected between the groups in any of the fall-related outcomes.

**Table 2.** Intention-to-treat analysis of changes in fall-related parameters, physical function, and body composition from baseline to 12 weeks.

Measures	AT+EX group^a^ (n=15)				AT-only group^b^ (n=15)				AT+EX vs AT-only	
	Baseline, mean (SD)	12 weeks, mean (SD)	Δ (12 weeks to baseline), mean (SD)	*P* value^c^	Baseline, mean (SD)	12 weeks, mean (SD)	Δ (12 weeks to baseline), mean (SD)	*P* value^c^	*P* value for baseline^d^	*P* value for 12 weeks to baseline^e^
Fall-related parameters										
ABC^f^ (score)	56.5 (17.8)	72.0 (13.6)	15.5 (15.7)	.002	57.9 (18.2)	68.0 (21.4)	10.2 (20.2)	.07	>.99	.43
FES-I^g^ (score)	29.5 (8.2)	24.4 (5.3)	–5.1 (6.1)	.01	30.4 (10.3)	24.9 (8.1)	–5.5 (8.0)	.01	.92	.90
SGDS^h^ (score)	4.5 (3.6)	2.5 (2.6)	–2.0 (2.3)	.006	5.4 (4.5)	4.0 (4.4)	–1.4 (2.1)	.02	.54	.46
MoCA^i^ (score)	23.8 (2.8)	24.7 (3.0)	0.9 (2.1)	.10	23.9 (3.2)	24.4 (2.7)	0.5 (2.4)	.49	.90	.59
Physical function										
Grip strength (kg)	22.0 (6.4)	23.6 (6.1)	1.5 (4.0)	.14	22.5 (5.2)	24.3 (5.1)	1.7 (3.7)	.12	.82	.90
SPPB^j^ (score)	10.3 (1.7)	11.5 (1.1)	1.1 (1.1)	.004	9.9 (2.2)	10.7 (1.5)	0.8 (1.7)	.09	.52	.23
Timed Up and Go (seconds)	9.7 (2.6)	8.3 (2.2)	–1.4 (2.1)	.008	9.9 (2.2)	8.7 (1.3)	–1.3 (1.3)	.002	.52	.81
Gait speed (m/s)	0.81 (0.23)	0.91 (0.15)	0.10 (0.23)	.11	0.79 (0.21)	0.85 (0.13)	0.06 (0.14)	.12	.84	.56
2-Minute Step Test (n)	156.0 (35.9)	182.7 (34.3)	26.7 (27.4)	.001	131.7 (41.2)	142.1 (36.3)	10.5 (32.3)	.10	.11	.10
Body composition										
BMI (kg/m²)	22.8 (2.7)	22.6 (2.8)	–0.3 (0.6)	.08	24.9 (3.0)	24.9 (3.0)	0.0 (0.5)	.97	.06	.15
Skeletal muscle index (kg/m²)	6.6 (0.8)	6.5 (0.8)	–0.1 (0.2)	.26	7.0 (0.9)	6.8 (0.9)	–0.2 (0.3)	.07	.16	.36
Total fat mass (kg)	17.2 (4.6)	16.8 (4.7)	–0.4 (1.0)	.09	20.8 (4.8)	20.5 (4.6)	–0.3 (0.9)	.97	.048	.17
Total lean mass (kg)	36.1 (6.0)	35.7 (6.3)	–0.4 (0.8)	.04	39.5 (6.9)	39.0 (6.3)	–0.5 (1.3)	.19	.17	.96

a AT+EX group: activity tracker and exercise group.

b AT-only group: activity tracker alone group.

c *P* values represent the differences between baseline and 12 weeks at each group using the paired 2-tailed *t* test or the Wilcoxon signed rank test.

d *P* values represent the differences between the AT+EX group and the AT-only group at baseline using the independent 2-tailed *t* test or the Mann-Whitney test.

e *P* values represent the comparison of changes between 2 groups using the independent 2-tailed *t* test or the Mann-Whitney test.

f ABC: Activities-Specific Balance Confidence.

g FES-I: Falls Efficacy Scale-International.

h SGDS: Short Geriatric Depression Scale.

i MoCA: Montreal Cognitive Assessment.

j SPPB: Short Physical Performance Battery.

### Physical Function

Regarding physical function, grip strength showed no significant change in both groups (AT+EX: 1.5 kg; *P*=.14 and AT-only: 1.7 kg; *P*=.12). The SPPB total score showed significant improvement in the AT+EX group (1.1 points; *P*=.004) but was not significant in the AT-only group (0.8 points; *P*=.09). The AT+EX group also demonstrated significant improvements in both the TUG test (1.4 seconds; *P*=.008) and the 2MST (26.7 steps;, *P*=.001), while the AT-only group showed a significant improvement only in the TUG test (1.3 seconds; *P*=.002). No significant differences were observed between the groups in terms of changes in any physical functional variables.

### Body Composition

Regarding body composition, the AT+EX group showed reductions, including a significant decrease in total lean mass by 0.4 kg (*P*=.04). In contrast, the AT-only group showed nonsignificant decreases in total lean mass and skeletal muscle index (*P*=.19 and *P*=.07, respectively). No significant differences between the groups were observed in the changes of any body composition variables.

### Changes in Mean Daily Steps

**Figure 2.**
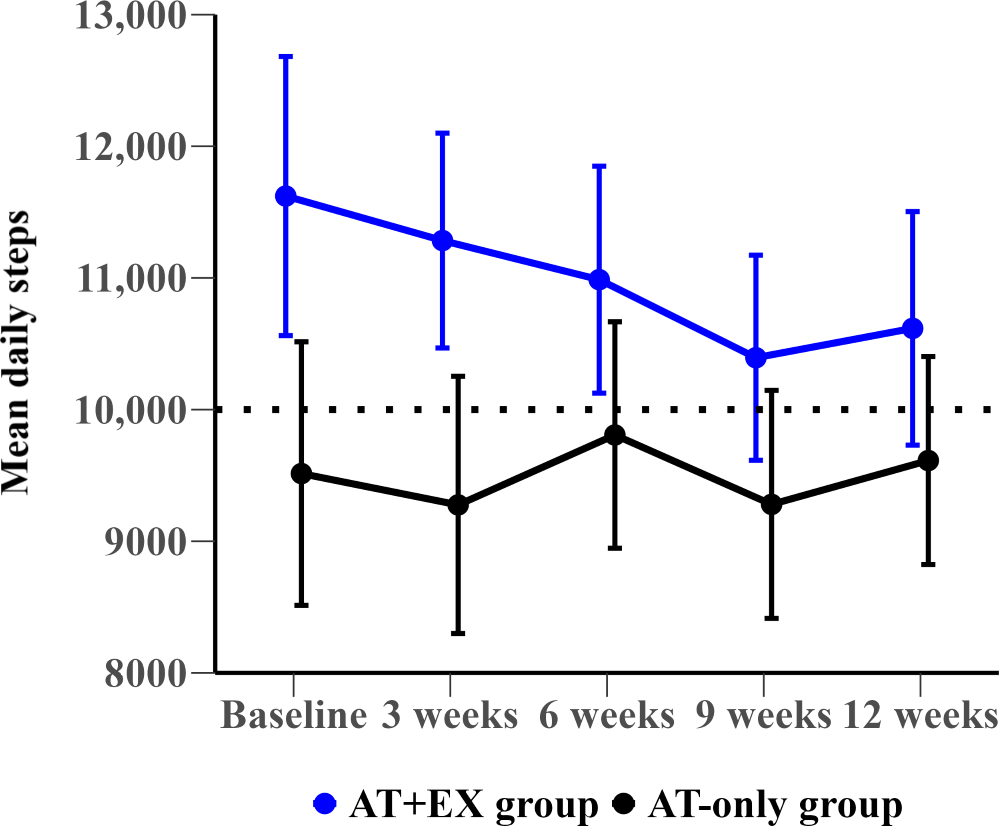
Mean daily steps with SE of the mean at baseline and over specific weeks (1, 3, 6, 9, and 12) for the total group. AT+EX group: activity tracker and exercise group; AT-only group: activity tracker alone group.

Figures2  3 present the mean daily steps, while Table 3 details changes in mean daily steps over 12 weeks for both the overall group and the subgroup with low physical performance. At baseline, the AT+EX group averaged 11,622 daily steps compared to 9514 steps in the AT-only group, showing a mean difference of 2108 steps (95% CI –691 to 4906; *P*=.14). Throughout the 12-week study period, both groups maintained high levels of daily steps, and differences between them were not statistically significant. Over the study period, the AT+EX group decreased by 1005 (from 11,622 to 10,617) steps, whereas the AT-only group increased by 98 (from 9514 to 9613) steps.

**Figure 3.**
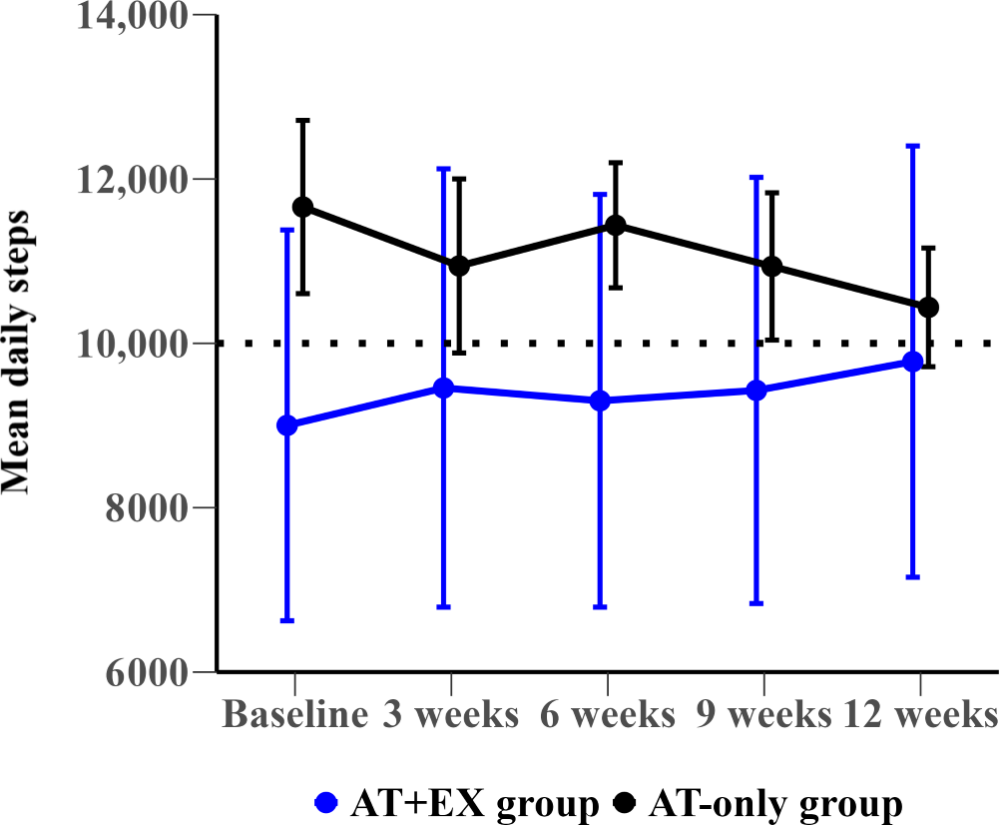
Mean daily steps with SE of the mean at baseline and over specific weeks (1, 3, 6, 9, and 12) for the low physical performance subgroup. AT+EX group: activity tracker and exercise group; AT-only group: activity tracker alone group.

**Table 3.** Differences in changes in mean daily step count over 12 weeks between groups for the total group and low physical performance subgroup.

	AT+EX group^a^, mean (SD)	AT-only group^b^, mean (SD)	Mean difference (95% CI)	*P* value^c^
Total study participants, n	15	15	—^d^	—
Mean daily steps				
Baseline	11,622 (4104)	9514 (3878)	2108 (–691 to 4906)	.14
Week 3	11,284 (3161)	9276 (3785)	2007 (–461 to 4476)	.11
Week 6	10,987 (3337)	9807 (3331)	1179 (–1192 to 3550)	.32
Week 9	10,486 (3013)	9280 (3352)	1206 (–1074 to 3486)	.29
Week 12	10,617 (3437)	9613 (3060)	1004 (–1318 to 3325)	.39
Changes in the steps from baseline				
Week 3 to baseline	–338 (2221)	–238 (1563)	–100 (–1537 to 1336)	.88
Week 6 to baseline	–635 (1885)	293 (1633)	–928 (–2248 to 392)	.15
Week 9 to baseline	–1136 (2493)	–235 (2021)	–902 (–2599 to 796)	.27
Week 12 to baseline	–1005 (2860)	98 (2870)	–1104 (–3247 to 1039)	.28
Subgroup of participants with low physical performance (SPPB^e^ scores ≤9), n	4	5	—	—
Mean daily steps				
Baseline	9002 (4755)	11,659 (2356)	–2657 (–7457 to 2143)	.31
Week 3	9457 (5023)	10,942 (2368)	–1485 (–6495 to 3525)	.57
Week 6	9301 (5186)	11,437 (1701)	–2137 (–6995 to 2722)	.41
Week 9	9427 (5248)	10,936 (2003)	–1509 (–6534 to 3516)	.57
Week 12	9778 (5334)	10,438 (1614)	–660 (–5610 to 4290)	.80
Changes in the steps from baseline				
Week 3 to baseline	455 (565)	–717 (1039)	1172 (–205 to 2549)	.08
Week 6 to baseline	298 (683)	–222 (1498)	520 (–1411 to 2451)	.54
Week 9 to baseline	425 (790)	–723 (1448)	1148 (–772 to 3068)	.2
Week 12 to baseline	776 (612)	–1221 (2060)	1997 (–553 to 4547)	.11

a AT+EX group: activity tracker and exercise group.

b AT-only group: activity tracker alone group.

c *P* value for comparisons between 2 groups calculated using a mixed model. Mean daily steps were calculated over specific weeks. Baseline steps were the average of the first week (days 1‐7). Subsequent steps were averaged between exercise sessions (weeks 1, 3, 6, and 9), and from week 9 to the final visit.

d Not applicable

e SPPB: Short Physical Performance Battery.

In the subgroup with low physical performance (SPPB scores ≤9), the AT+EX group started with 9002 steps, lower than the AT-only group’s 11,659 steps, with a mean difference of 2657 steps (95% CI –7457 to 2143; *P*=.31). By week 12, the AT+EX subgroup increased by 776 steps, while the AT-only subgroup decreased by 1221 steps. However, the difference between the groups remained nonsignificant (mean difference 1997 steps, 95% CI –553 to 4547; *P*=.11).

## Discussion

### Principal Findings

We investigated the effects of integrating activity trackers with a home-based multicomponent exercise intervention (AT+EX group) compared to using activity trackers alone (AT-only group) in older adults (≥60 years) with a history of falls and COVID-19. Our results showed that both groups exhibited significant improvements in fear of falling (FES-I score) and depression (SGDS) as well as enhanced physical function (TUG test). Notably, the AT+EX group showed improvements in the ABC score, SPPB score, and 2MST. However, no significant between-group differences were observed in any outcome variables. Although there is no consensus on the cutoff value of the ABC score for predicting future falls, previous studies have suggested cutoff values of <58‐67 [36,37]. In this study, the mean ABC score at baseline was 56.5 (SD 17.8) and 57.9 (SD 18.2) in the AT+EX group and AT-only group, respectively. After the intervention, the ABC score improved significantly in the AT+EX group (15.5 points; *P*=.002) but was not significant in the AT-only group (10.2 points; *P*=.07). An increase in the ABC score reflects a reduction in fear of falling and activity limitation as well as an improvement in the quality of life of the study participants [38]. We also showed that the SPPB score improved significantly in the AT+EX group (1.1 points; *P*=.004) but was not significant in the AT-only group (0.8 points; *P*=.09) after the intervention. Better physical performance reduces the risk of mobility-related disability [32]. Although no significant increase in daily steps was observed in either group, the participants maintained a high activity level of approximately 10,000 steps per day over the 12-week study period.

To date, few trials have evaluated the impact of activity trackers on fall-related outcomes, and the results have been inconsistent. Yamada et al [23] reported that a 6-month pedometer-based behavioral change program effectively improved fear of falling, physical activity, locomotive function, and leg muscle mass compared with the corresponding in the control group (*P*<.05). Another study by Oliveira et al [22] examined the effectiveness of a fall prevention intervention with a Fitbit (Google Inc). Although mobility goal attainment improved at 6 months compared to that in the control group, no between-group differences were noted in fall rates, fear of falling, physical activity, and mobility limitation at 6 or 12 months.

Our study showed significant improvements in fear of falling and depression in both the AT+EX and AT-only groups without significant between-group differences. However, we did not observe any improvements in cognitive function. This may be attributed to the fact that our study included participants with normal cognitive function, which may have underestimated the effect on cognitive function. Given the high prevalence of depression and cognitive impairment and the close interaction between 2 common disorders among older adults [39], it would be useful to conduct future large clinical trials to demonstrate the effectiveness of exercise in patients with these conditions.

Previous studies have compared groups using activity trackers to control groups with no intervention, whereas our study compared the AT+EX group to the AT-only group. Moreover, these studies did not include direct exercise training in their interventions. Although cognitive decline and mood disorders, such as depression and anxiety, are associated with fear of falling, research on how interventions affect these variables is lacking [40,41]. Considering the effectiveness of exercise in improving physical and cognitive functions, further research is needed to explore the impact of integrating activity trackers with home-based exercise interventions on cognitive and mood parameters as well as the fear of falling.

### Comparison With Prior Work

Studies on the impact of wearable devices on physical function in older adults are limited and have shown mixed results [22,23,42-48]. A few studies using pedometers or Fitbit devices reported improvements in physical function [42,44,47,48]. For instance, 2 randomized controlled trials with pedometer-based walking programs have found significant improvements in walking speed and physical performance [47,48]. Another study using Fitbit-based interventions showed improvements in the 30-second chair stand test and physical endurance [42,44]. However, some studies using wearable devices have not shown improvements in physical function [43,45,46].

In our study, both groups showed overall improvements in physical function, although no statistically significant differences were observed between the groups. However, the AT+EX group showed significant improvements in the total SPPB score, exceeding the clinically significant threshold of a 1-point improvement [31]. Moreover, the AT+EX group showed significant improvements in mobility and endurance, as measured by the TUG test and 2MST. These findings suggest that a multicomponent exercise intervention may be more effective in enhancing physical function (ie, balance, gait, mobility, and endurance) in older adults than using activity trackers alone.

The improvement in fear of falls and physical function in both groups using activity trackers may be related to the amount of physical activity. Physical activity has been associated with a lower risk of falls and improved physical function in older adults [49]. A comprehensive analysis of systematic reviews showed that the use of activity trackers is effective in increasing physical activity levels across various age groups [50]. However, we did not observe any increase in daily step counts in either the AT+EX or AT-only group over the 12-week period. There are several potential reasons for this lack of increase. First, the participants’ baseline activity level was already high, and the target step count was set at a maximum of 10,000 steps per day. Those who met this goal were encouraged to sustain their activity level rather than increase it further. Second, the reactivity effect may have influenced the results; participants may have initially increased their activity levels upon realizing that their physical activity was monitored [51].

Although no significant increases were observed in daily step counts, both groups maintained close to 10,000 steps per day throughout the 12 weeks. Notably, even the AT-only group, which did not receive additional feedback, showed a consistently high step count. This suggests that setting a specific step goal and synchronizing the activity tracker data with the app provided participants with daily visual feedback, allowing them to objectively monitor their physical activity levels and maintain their daily step counts. However, considering that this study was conducted over 12 weeks, further long-term studies are required to determine the sustained effectiveness of activity trackers without additional interventions. Moreover, a subgroup analysis revealed that participants with low physical performance (SPPB scores ≤9) exhibited increased daily step counts in the AT+EX group. This indicates that the effectiveness of activity tracker use may vary based on participants’ physical performance. Further research is required to identify the subgroups that benefit the most from using activity trackers.

Regarding body composition, the AT+EX group showed an unexpected substantial decrease in lean body mass. This may be attributed to the lack of nutritional intervention in this study. Inadequate nutritional support for increased physical activity may result in reductions in both fat and muscle mass [52]. Moreover, the multicomponent exercise program focused on enhancing balance and physical function rather than increasing muscle mass. Considering the inadequate protein intake of older people in Korea [53], future research should include both physical activity and nutritional interventions including protein supplementation. Further studies are required to determine the most effective exercise regimens when combined with activity trackers.

### Limitations

This study had several limitations. First, the study duration was only 12 weeks, which may not have been sufficient to observe the long-term effects of the intervention. Second, the sample size was relatively small, potentially limiting the generalizability of the results to individuals who are not motivated to increase their physical activity levels. Third, the participants were already relatively active at baseline, which could have constrained the observable benefits of increased physical activity. Fourth, the study lacked a control group with no intervention, which restricted the possibility of comparing intervention effects. Fifth, participants’ nutritional intake and other lifestyle changes were not controlled.

### Conclusions

Although no significant between-group differences were observed, both activity trackers with and without a home-based multicomponent exercise program effectively improved fall-related parameters (fear of falling and depression) and physical function in older adults compared to baseline. Our findings underscore the potential of digital health technologies to enhance fall prevention strategies.

## Supplementary material

10.2196/64458 Multimedia Appendix 1 Exercise training protocol.

10.2196/64458 Checklist 1 CONSORT (Consolidated Standards of Reporting Trials) checklist.

## Abbreviations

2MST: 2-Minute Step Test
ABC: Activities-Specific Balance Confidence
AT+EX group: activity tracker and exercise group
AT-only group: activity tracker alone group
CONSORT: Consolidated Standards of Reporting Trials
FES-I: Falls Efficacy Scale-International
SGDS: Short Geriatric Depression Scale
SPPB: Short Physical Performance Battery
TUG: Timed Up and Go
