# Supplementary material for: Effects of Integrating Wearable Activity Trackers With a Home-Based Multicomponent Exercise Intervention on Fall-Related Parameters and Physical Function in Older Adults: Randomized Controlled Trial
Source: JMIR Mhealth Uhealth. 2025 May 8;13:e64458. doi: 10.2196/64458 (PMC12080971; doi:10.2196/64458)
Supplement: Multimedia Appendix 1 [file mhealth-v13-e64458-s001.docx]

| **First stage (weeks 1-4)** | **Second stage (weeks 5-8)** | | | **Third stage (weeks 9-12)** |  |
| --- | --- | --- | --- | --- | --- |
| **Balance exercise**  Sifting weight back and forth with support Side leg raise with support One-legged stance with support | **Balance exercise** Sifting weight back and forth with support  Walking in a straight line (forward, backward)  Walking on the toes  One-legged stance with head moving | | | **Balance exercise** Sifting weight back and forth without support  Walking on the toes with eyes variations  Walking in a straight line with eyes variations |  |
|  |  |  |  |  |  |
|  |  |  |  |  |  |
|  |  |  |  |  |  |
|  |  |  |  |  |  |
| **Strength training**  Band (color: red) in a sitting position (10 rep, 2 set) Latissimus dorsi (pull-down) Chest (chest press) Leg (left/right leg extension and plantar flexion) | | **Strength training**  Band (color: red/green) in a sitting position Latissimus dorsi (lowing) Arms (sword fighter)  Hip (abduction exercise)  Leg (left/right leg press) | **Strength training**  Band (color: red/green) in a sitting position Latissimus dorsi (pull-down) Shoulders (front and lateral raise)  Hip (abduction exercise)  Leg (left/right leg extension and plantar flexion) | | |
|  |  |  |  |  |  |
|  |  |  |  |  |  |
|  |  |  |  |  |  |
|  |  |  |  |  |  |
|  |  |  |  |  |  |
|  |  |  |  |  |  |
| **Gait and aerobic training**  Walking in place  Running in place Knee up | **Gait and aerobic training** Step ups (step box: 10cm)  Sidestep (step box: 10cm)  Step in and out (step box: 10cm) | | | **Gait and aerobic training** Step ups with eyes fixed (step box: 10cm)  Sidestep with eyes fixed (step box: 10cm)  Single leg backwards (step box: 10cm) |  |
|  |  |  |  |  |  |
|  |  |  |  |  |  |
|  |  |  |  |  |  |
|  |  |  |  |  |  |
| **Dual-task training**  Saying the name of the fruit while walking in place  Wrist fold with one hand and counting numbers with the opposite hand (left/right)  Sitting exercises (moving arms and legs in opposite directions) | | **Dual-task training** Saying the name of the animal while walking in place Wrist fold with one hand and drawing a circle with the opposite hand (left/right) Standing exercises (moving arms and legs in opposite directions) | **Dual-task training**  Saying the name of the country while walking in place Wrist fold with one hand and drawing a square with the opposite hand (left/right)  One-legged stance with finger folding | | |
|  |  |  |  |  |  |
|  |  |  |  |  |  |
|  |  |  |  |  |  |
